# Fully automatic segmentation and objective assessment of atrial scars for long‐standing persistent atrial fibrillation patients using late gadolinium‐enhanced MRI

**DOI:** 10.1002/mp.12832

**Published:** 2018-03-15

**Authors:** Guang Yang, Xiahai Zhuang, Habib Khan, Shouvik Haldar, Eva Nyktari, Lei Li, Ricardo Wage, Xujiong Ye, Greg Slabaugh, Raad Mohiaddin, Tom Wong, Jennifer Keegan, David Firmin

**Affiliations:** ^1^ Cardiovascular Research Centre Royal Brompton Hospital London SW3 6NP UK; ^2^ National Heart and Lung Institute Imperial College London London SW7 2AZ UK; ^3^ School of Data Science Fudan University Shanghai 201203 China; ^4^ Department of Biomedical Engineering Shanghai Jiao Tong University Shanghai 200240 China; ^5^ School of Computer Science University of Lincoln Lincoln LN6 7TS UK; ^6^ Department of Computer Science City University London London EC1V 0HB UK

**Keywords:** atrial fibrillation, cardiovascular magnetic resonance imaging, late gadolinium‐enhanced MRI, medical image segmentation, whole heart segmentation

## Abstract

**Purpose:**

Atrial fibrillation (AF) is the most common heart rhythm disorder and causes considerable morbidity and mortality, resulting in a large public health burden that is increasing as the population ages. It is associated with atrial fibrosis, the amount and distribution of which can be used to stratify patients and to guide subsequent electrophysiology ablation treatment. Atrial fibrosis may be assessed noninvasively using late gadolinium‐enhanced (LGE) magnetic resonance imaging (MRI) where scar tissue is visualized as a region of signal enhancement. However, manual segmentation of the heart chambers and of the atrial scar tissue is time consuming and subject to interoperator variability, particularly as image quality in AF is often poor. In this study, we propose a novel fully automatic pipeline to achieve accurate and objective segmentation of the heart (from MRI Roadmap data) and of scar tissue within the heart (from LGE MRI data) acquired in patients with AF.

**Methods:**

Our fully automatic pipeline uniquely combines: (a) a multiatlas‐based whole heart segmentation (MA‐WHS) to determine the cardiac anatomy from an MRI Roadmap acquisition which is then mapped to LGE MRI, and (b) a super‐pixel and supervised learning based approach to delineate the distribution and extent of atrial scarring in LGE MRI. We compared the accuracy of the automatic analysis to manual ground truth segmentations in 37 patients with persistent long‐standing AF.

**Results:**

Both our MA‐WHS and atrial scarring segmentations showed accurate delineations of cardiac anatomy (mean Dice = 89%) and atrial scarring (mean Dice = 79%), respectively, compared to the established ground truth from manual segmentation. In addition, compared to the ground truth, we obtained 88% segmentation accuracy, with 90% sensitivity and 79% specificity. Receiver operating characteristic analysis achieved an average area under the curve of 0.91.

**Conclusion:**

Compared with previously studied methods with manual interventions, our innovative pipeline demonstrated comparable results, but was computed fully automatically. The proposed segmentation methods allow LGE MRI to be used as an objective assessment tool for localization, visualization, and quantitation of atrial scarring and to guide ablation treatment.

**Abbreviation**
AF: atrial fibrillation
LA: left atrium
PV: pulmonary veins
SVM: support vector machine
CV: cross‐validation
ROI: region of interest
LGE‐MRI: late gadolinium‐enhanced magnetic resonance imaging

## 1 Introduction

Atrial fibrillation (AF) is the most common arrhythmia of clinical significance, resulting in a large public health burden that is increasing as the population ages. It occurs when chaotic and disorganized electrical activity develops in the atria, causing muscle cells to contract irregularly and rapidly. It is associated with structural remodeling, including fibrotic changes in the left atrium^1^ and can cause increased morbidity, especially stroke and heart failure. It also results in poor mental health, dementia, and increased mortality, costing the NHS £2.2 billion per year.^2^

The electrical impulses that trigger AF frequently originate in the pulmonary veins (PV). Radio frequency ablation treatment aims to eliminate AF by electrically isolating the PV. However, the success rate for a single catheter ablation procedure is just 30–50% at 5‐yr follow‐up^3^, ^4^ and multiple ablations are frequently required. Accurate knowledge of native and previous ablation scarring could be used to stratify patients and guide ablation treatment, ultimately reducing the need for repeat procedures.^5^

The current clinical gold standard for assessment of atrial scarring is electro‐anatomical mapping (EAM), performed during an electrophysiological (EP) study.^6^ However, this is an invasive technique which uses ionizing radiation and the accuracy is suboptimal, with reported errors of up to 10 mm in the localization of scar tissue.^7^, ^8^

Late gadolinium enhancement (LGE) magnetic resonance imaging (MRI) is an established noninvasive technique for detecting myocardial scar tissue.^9^ With this technique, healthy and scar tissues are differentiated by their altered wash‐in and wash‐out contrast agent kinetics, which result in scar tissue being seen as a region of enhanced or high signal intensity while healthy tissue is “nulled.” Atrial 3D LGE MRI has been used to assess patient suitability for AF ablation by identifying potential nonresponders,^10^, ^11^, ^12^, ^13^, ^14^, ^15^, ^16^ and to define the most appropriate ablation approach.^12^, ^13^, ^17^ In addition, visualization and quantitation of native and postablation atrial scarring derived from LGE MRI has been used to guide initial and follow‐up ablation procedures.^13^, ^14^, ^18^, ^19^, ^20^, ^21^ Histopathological studies in pigs have validated LGE MRI for the characterization of AF ablation‐induced wall injury.^22^

Visualization and quantification of atrial scarring requires objective, robust, and accurate segmentation of the enhanced scar regions from the LGE MRI images. Essentially, there are two segmentations required: one showing the cardiac anatomy (geometry), particularly the LA wall and PV, the other delineating the enhanced scar regions. The former segmentation is required to rule out confounding enhanced tissues from other parts of the heart, for example, the mitral valve and aorta, or the enhancement from nonheart structures while the latter is a prerequisite for visualization and quantitation. Segmentation of the atrial scarring from LGE MRI images is a very challenging problem. Firstly, the LA wall is very thin and scarring is hard to distinguish even by experienced expert cardiologists specialized in cardiac MRI. Secondly, residual respiratory motion, heart rate variability, low signal‐to‐noise ratio (SNR), and contrast agent wash‐out during the long acquisition (current scanning time ≈10 min) frequently result in image quality being poor. Artifactual enhanced signal from surrounding tissues may also result in a large number of false positives.

A grand challenge for evaluation and benchmarking of various atrial scarring segmentation methods has shown promising results^5^ although most have relied on manual segmentation of the LA wall and PV. This has several drawbacks: (a) it is a time‐consuming task; (b) there are intra‐ and interobserver variations; and (c) it is less reproducible for a multicenter and multiscanner study. Moreover, a number of studies have assumed a fixed thickness of the LA wall although there is no evidence that this is the case. Depending on the actual wall thickness, subsequent reorientation and interpolation of the MR images results in varying partial volume effects, which affect the apparent thickness of the LA wall. Inaccurate manual segmentation of the LA wall and PV can further complicate the delineation of the atrial scarring and its quantitation can be error prone.

The LA and PV would ideally be segmented from the cardiac and respiratory‐gated LGE MRI dataset. However, this is difficult as the inversion magnetization preparation used reduces the blood pool signal and normal myocardium is nulled. Other options are to segment them from a separately acquired breath‐hold magnetic resonance angiogram (MRA) study^15^, ^23^, ^24^ or from a respiratory and cardiac gated 3D balanced steady‐state‐free precession (b‐SSFP) “Roadmap” study.^25^ While breath‐hold MRA shows the LA and PV with high contrast, these acquisitions are generally ungated and acquired in an inspiratory breath‐hold. The anatomy extracted can therefore be highly deformed compared to that in the LGE MRI study. Although the gated 3D Roadmap acquisition takes longer to acquire, it is in the same respiratory phase as the LGE MRI and the extracted anatomy can be better matched. Cardiac anatomy has previously been defined by atlas‐based segmentation of MRA^24^ and by using a statistical shape model^25^ on 3D Roadmap data. Table 1 provides a summary of previously published methods on atrial scarring segmentation using LGE MRI and our proposed method.

**Table 1:** Summary of the previously published methods for atrial scarring segmentation and our proposed method

References	Subjects (number)	Cardiac anatomy segmentation (modality)	Atrial scarring segmentation	Evaluation of atrial scarring segmentation (results: mean ± std)
Oakes et al.^10^	Human (81)	Manual segmentation of LA wall (LGE MRI)	2–4 SD	Atrial scarring percentage (8 ± 4, 21 ± 6, 50 ± 15)^a^
Knowles et al.^23^	Human (7)	Semi‐automatic thresholding and region growing (MRA)	Maximum intensity projection	Atrial scarring percentage (31 ± 10)^b^
Perry et al. (2012)^68^	Human (34)	Manual segmentation of LA wall (LGE MRI)	k‐means Clustering	Dice (81 ± 11, ground truth by manually selected thresholds)
Ravanelli et al.^15^	Human (10)	Manual segmentation of LA and PV in 3D (MRA)	4 SD	Dice (60 ± 21 ground truth by a semi‐automatic approach)^c^
Karim et al.^25^	Human (15)	Statistical shape model with manual correction (b‐SSFP)	Graph cuts	Dice, ROC, and total scar volume^d^
Tao et al.^24^	Human (46)	Automatic atlas‐based method with level set refinement (MRA)	Maximum intensity projection	Qualitative visualization (N/A)
Proposed method	Human (37)	Fully automated multiatlas whole heart segmentation (b‐SSFP)	Super‐pixel and SVM	Multiple quantitative metrics (Dice: 79 ± 5)

a Results (%) for mild (n = 43), moderate (n = 30), and extensive (n = 8) enhancement cases.

b Moderate and extensive enhancement cases.

c The Dice score was calculated for an automated atrial scarring segmentation. The method was also evaluated using Bland–Altman analysis of the atrial scarring percentage (after skeletonization) obtained from LGE MRI and EAM.

d Multiple Dice scores were calculated for various experimental settings, and they were reported by plotting the median Dice scores (around 80) with the minimum and the maximum.

We propose a novel fully automatic segmentation and objective assessment of atrial scarring for long‐standing persistent AF patients scanned by LGE MRI. The LA chamber and PV are defined using a multiatlas‐based whole heart segmentation (MA‐WHS) method on b‐SSFP Roadmap MRI images. LA and PV geometry is resolved by mapping the segmented Roadmap anatomy to LGE MRI using the DICOM header data and is further refined by affine and nonrigid registration steps. LGE MRI images are segmented by a novel Simple Linear Iterative Clustering (SLIC)‐based super‐pixels method. A support vector machine (SVM)‐based supervised classification is then applied to segment the atrial scarring within the segmented LA and PV geometry. In this study, two validation steps have been performed — one for the LA chamber and PV segmentation and one for the atrial scarring segmentation — both against established ground truth from manual segmentations by experienced expert cardiologists specialized in cardiac MRI.

## 2 Materials and methods

### 2.A. Data acquisition

Cardiac MR data were acquired on a Siemens Magnetom Avanto 1.5T scanner (Siemens Medical Systems, Erlangen, Germany).

Transverse navigator‐gated 3D LGE MRI^10^, ^26^, ^27^ was performed using an inversion prepared segmented gradient echo sequence (TE/TR 2.2 ms/5.2 ms) 15 min after gadolinium (Gd) administration (Gadovist — gadobutrol, 0.1 mmol/kg body weight, Bayer‐Schering, Berlin, Germany).^28^ The inversion time was set to null the signal from normal myocardium. Detailed scanning parameters are: 30–34 slices at 1.5 × 1.5 × 4 mm^3^, reconstructed to 60–68 slices at 0.75 × 0.75 × 2 mm^3^, field‐of‐view 380 × 380 mm^2^, acceleration factor of 2 using generalized autocalibrating partially parallel acquisition (GRAPPA), acquisition window 125 ms positioned within the subject‐specific rest period, single R‐wave gating, chemical shift fat suppression, flip angle 20°. Data were acquired during free‐breathing using a crossed‐pairs navigator positioned over the dome of the right hemi‐diaphragm with navigator acceptance window size of 5 mm and CLAWS respiratory motion control.^29^ The nominal acquisition duration was 204–232 cardiac cycles assuming 100% respiratory efficiency.

Prior to contrast agent administration, coronal navigator‐gated 3D b‐SSFP (TE/TR 1/2.3 ms) Roadmap data were acquired with the following parameters: 80 slices at 1.6 × 1.6 × 3.2 mm^3^, reconstructed to 160 slices at 0.8 × 0.8 × 1.6 mm^3^, field‐of‐view 380 × 380 mm^2^, acceleration factor of 2 using GRAPPA, partial Fourier 6/8, acquisition window 125 ms positioned within the subject‐specific rest period, chemical shift fat suppression, flip angle 70°. Off resonant blood from the lungs arriving in the LA and PV can result in signal loss,^30^ which in our application, is minimized by using the shortest TE/TR possible. This was achieved by using nonselective RF excitation.^31^ Data were acquired during free‐breathing using a crossed‐pairs navigator positioned over the dome of the right hemi‐diaphragm with navigator acceptance window size of 5 mm and CLAWS respiratory motion control.^29^ The nominal acquisition duration was 241 cardiac cycles assuming 100% respiratory efficiency.

### 2.B. Patients

In agreement with the local regional ethics committee, cardiac MRI was performed in long‐standing persistent AF patients between 2011 and 2013. Gadolinium administration is contraindicated in patients with severe kidney disease due to an increased, but still rare, risk of developing nephrogenic systemic fibrosis. Consequently, LGE MRI was not performed in any patient with 30 ml/min/1.73 m^2^.

The image quality of each dataset was scored by a senior cardiac MRI physicist on a Likert‐type scale — 0 (non‐diagnostic), 1 (poor), 2 (fair), 3 (good), and 4 (very good) — depending on the level of SNR, appropriate TI, and the existence of navigator beam and ghost artifacts. Thirty‐seven cases with image quality greater or equal to 2 were retrospectively entered into this study including 11 preablation (included ~65% of preablation cases) and 26 postablation scans (included ~92% of post‐ablation cases).

### 2.C. Multiatlas whole heart segmentation (MA‐WHS)

**Figure 1:**
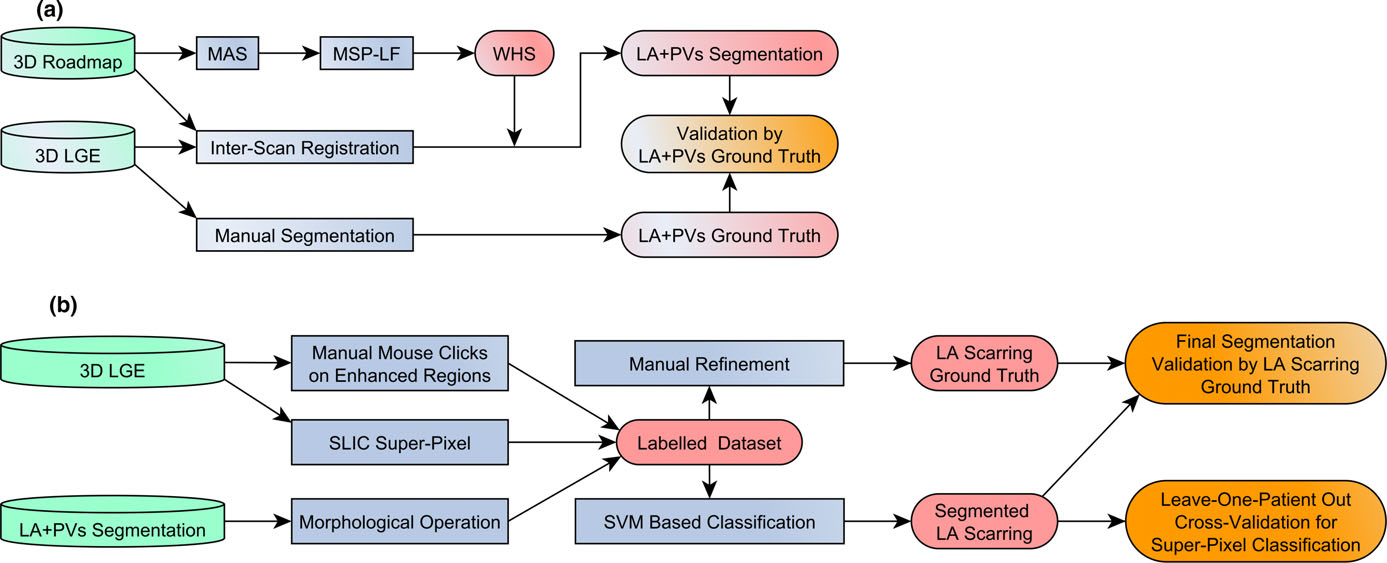
(a) Flowchart of the LA + PV segmentation via MA‐WHS and its validation. (b) Flowchart of the fully automatic atrial scarring segmentation including atrial scarring ground truth construction, super‐pixel and SVM classification‐based segmentation and leave‐one‐patient‐out cross‐validation. Abbreviations: LA + PV, left atrium and pulmonary veins; MAS, multiatlas propagation‐based segmentation; MSP‐LF, multiscale patch‐based label fusion; WHS, whole heart segmentation. [Color figure can be viewed at http://wileyonlinelibrary.com]

A multiatlas approach^32^, ^33^ was developed to derive the whole heart segmentation of the Roadmap acquisition, which was then mapped to LGE MRI [Fig. 1(a)]. This segmentation consists of two major steps: (a) atlas propagation based on image registration algorithms and (b) label fusion from multiatlas‐propagated segmentation results.

First we obtained 30 MRI Roadmap studies from the Left Atrium Segmentation Grand Challenge organized by King's College London^34^ together with manual segmentations of the left atrium, pulmonary veins, and appendages. In these, we further labeled the right and left ventricles, the right atrium, the aorta and the pulmonary artery, to generate 30 whole heart atlases. These 30 MRI Roadmap studies were employed only for building an independent multiatlas dataset, which is then used for segmenting our Roadmap studies that are linked with the LGE MRI scans for the AF patients.

Let I be the target image to be segmented, $\left\{\left(A_{a},L_{a}\right)|a=1,\ldots N\right\}$ be the set of atlases, where N=30 *,* $A_{a}$ and $L_{a}$ are, respectively, the intensity image and corresponding segmentation label image of the *a*th atlas. For each atlas, MA‐WHS performs an atlas‐to‐target registration, by maximizing the similarity between the images, to derive the set of warped atlases,

(1) $$T_{a}=\arg\min_{T_{a}}\;\text{ImageSmilarity}\left(I,A_{a}\right),\text{and}\left\{\begin{array}{c} A_{a}=T_{a}\left(A_{a}\right)\\ L_{a}=T_{a}\left(L_{a}\right) \end{array}\right.,$$

in which $T_{a}$ is the resulting transformation of the registration and $\left\{\left(A_{a},L_{a}\right)|a=1,\ldots N\right\}$ are, respectively, the warped atlas intensity image and corresponding segmentation result. Here, we employ the hierarchical registration for segmentation propagation, which was specifically designed for the whole heart MRI images and consists of three steps, namely the global affine registration for localization of the whole heart, the local affine registration for the initialization of the substructures, and the fully deformable registration for local detail refinement.^35^ Image similarity metrics evaluate how similar the atlas and target image are. In this work, we propose to use the spatially encoded mutual information (SEMI) method, which has been shown to be robust against intensity nonuniformity and different intensity contrast,^36^ that is

(2) $$\text{ImageSimilarity}\left(I,A_{a}\right)=\left\{S_{1},\ldots ,S_{n_{s}}\right\}$$

where $\left\{S_{1},\ldots ,S_{n_{s}}\right\}$ are the SEMI and computed based on the spatially encoded joint histogram,

(3) $$H_{s}\left(I,A_{a}\right)=\sum_{x\in \Omega }w_{1}\left(I\left(x\right)\right)w_{2}\left(A_{a}\left(x\right)\right)W_{s}\left(x\right).$$

Here, $w_{1}\left(I(x)\right)$ and $w_{2}\left(A_{a}\left(x\right)\right)$ are Parzen window estimation and $W_{s}\left(x\right)$ is a weighting function to encode the spatial information.^36^

After the multiatlas propagation, a label fusion algorithm is required to generate one final segmentation of the LA from the 30 propagated results,

(4) $$L_{I}=\text{LabelFusion}\left(\left\{\left(A_{1},L_{1}\right)\ldots \left(A_{N},L_{N}\right)\right\}\right).$$

The label fusion decides how to combine the multiple classification results into one labeling result. Since the atlases can produce segmentations with dramatically different accuracy at different locations, it should evaluate the performance of each atlas locally and assigns different weights for the atlases at each pixel of the target image in decision fusion.

The recent literatures have many new methods^37^, ^38^, ^39^, ^40^, ^41^, ^42^, ^43^, ^44^ on improving multiatlas segmentation using sophisticatedly designed algorithms, which generally need to evaluate local similarity between patches from the atlases and the target image for local weighted label fusion,

(5) $$L_{I}\left(x\right)={\text{argmax}}_{l\in \{l_{\mathrm{bk}},l_{\mathrm{la}}\}}\sum_{a}w_{a}\left(S(I,A_{a},x)\right)\delta \left(L_{a}\left(x\right),l\right),$$

in which $l_{\mathrm{bk}}$ and $l_{\mathrm{la}}$ indicate the labels of the background and left atrium, respectively, and the local weight $w_{a}\left(\cdot \right)\propto S\left(\cdot \right)$ is determined by the local similarity S(·) between the target image and the atlas. δ(a,b) is the Kronecker delta function which returns 1 when a=b and returns 0 otherwise.

For the LA segmentation, we propose to use the multiscale patch‐based label fusion (MSP‐LF). This is because the intensity distribution of the blood pool in the LA is almost identical to that of the blood pool in the other chambers and great vessels. The multiscale space theory can handle different level information within a small patch and has been applied to feature extraction/detection and image matching.^33^, ^44^, ^45^, ^46^, ^47^, ^48^, ^49^, ^50^ The patches we compute from different scale spaces can represent the different levels of structural information, with low scale capturing local fine structure and high scale suppressing fine structure but providing global structural information of the image. This is different from the conventional patch‐based methods, which only compute the local structural information within the patch. To avoid increasing the computational complexity, we adopt the multiresolution implementation and couple it with the MSP where the high‐scale patch can be efficiently computed using a low‐resolution image space. The local similarity between two images using the MSP measure is computed, as follows,

(6) $$S_{\mathrm{msp}}\left(I,A_{a},x\right)=\sum_{s}S\left(I^{\left(s\right)},A_{a}^{\left(s\right)},x\right)$$

where $I^{\left(s\right)}=I*\text{Gaussian}\left(0,\sigma_{s}\right)$ is the target image from *s* scale space which is computed from the convolution of the target image with Gaussian kernel function with scale *s*. Here, we compute the local similarity in multiscale image using the conditional probability of the images,

(7) $$S\left(I^{\left(s\right)},A_{a}^{\left(s\right)},x\right)=p\left(i_{x}|j_{x}\right)=\frac{p(i_{x},j_{x})}{p(j_{x})}.$$

where $i_{x}=I^{\left(s\right)}\left(x\right)$ and $j_{x}=A_{a}^{\left(s\right)}\left(x\right)$ and the conditional image probability is obtained from the joint and marginal image probability which can be calculated using the Parzen window estimation.^51^

For each patient, the Roadmap dataset was then registered to the LGE MRI dataset using the DICOM header data, and then refined by affine and nonrigid registration steps.^36^ The resulting transformation was applied to the MA‐WHS‐derived cardiac anatomy to define the endocardial LA boundary and PV on the LGE MRI dataset for each patient. It is of note that both our Roadmap and LGE MRI data were cardiac gated and acquired in end expiration, which minimizes significant shape deformation between the two.

### 2.D. Atrial scarring segmentation

#### 2.D.1. Oversegmentation by simple linear iterative clustering (SLIC)‐based super‐pixels

We used a simple linear iterative clustering (SLIC)‐based super‐pixel method^52^ to oversegment LGE MRI images in order to separate potential enhanced atrial scarring regions from other tissues [Fig. 1(b)]. Super‐pixel algorithms group pixels into perceptually meaningful patches with similar size, which can be used to replace the regular pixel grid. Consequently, the derived super‐pixel patches can capture and mitigate image redundancy, and therefore provide a significant primitive from which image features can be calculated effectively and efficiently. In summary, super‐pixel methods have been proven to have following benefits: (a) super‐pixels can adhere well to perceptually meaningful object boundaries in images; (b) super‐pixels can reduce computational complexity of extracting image features; (c) for segmentation applications, super‐pixels can improve the performance while reducing the computation time.^53^ In this study, we proposed to use a SLIC‐based super‐pixel method, which has been successfully applied to solve various medical image analysis problems.^54^, ^55^ It has also demonstrated better segmentation accuracy and superior adherence to object boundaries, and it is faster and more memory efficient compared to other state‐of‐the‐art super‐pixels methods.^52^ Based on local k‐means clustering, the SLIC method iteratively groups pixels into super‐pixels. The clustering proximity is estimated in both intensity and spatial domains that is

(8) $$D=\sqrt{d_{c}^{2}+{\left(\frac{d_{s}}{S}\right)}^{2}m^{2}},$$

in which $d_{c}=\sqrt{{\left(I_{j}-I_{i}\right)}^{2}}$ measures the pixel intensity difference of a gray scale image and $d_{s}=\sqrt{{\left(x_{j}-x_{i}\right)}^{2}+{\left(y_{j}-y_{i}\right)}^{2}}$ describes the spatial distance between each pixel and the geometric center of the super‐pixel. SLIC is initialized by sampling the target slice of the LGE MRI image into a regular grid space with grid interval of S pixels. To speed up the iteration, SLIC limits the size of search region of similar pixels to 2S × 2S around the super‐pixel center (namely local k‐means clustering). In addition, parameter m balances the weighting between intensity similarity $d_{c}$ and spatial proximity $d_{s}$. In this study, we initialized S to 4 pixels that is 2.8 × 2.8 mm^2^ considering the LA wall thickness is approximately 3 mm,^56^, ^57^ and also take into account that the super‐pixel size is still large enough to extract statistics of the grouped pixel intensities. In addition, *m* was chosen by visual inspection of the oversegmented results, and it was fixed when the super‐pixel results adhered well with the LA wall boundary.

#### 2.D.2. Support vector machines (svm) based classification

After SLIC segmentation, we use Support Vector Machines (SVM) to classify the oversegmented super‐pixels into enhanced atrial scarring regions and nonenhanced tissues. SVM provide a powerful technique for supervised binary classification^58^ (refer to Supporting Material Appendix S1).

In order to train the SVM classifier, we built a training dataset containing enhanced and nonenhanced super‐pixel patches. This has been done by (a) an experienced expert cardiologist specialized in cardiac MRI performing manual mouse clicks to select the enhanced scar regions; (b) combining the mouse clicks and SLIC segmentation to label the enhanced super‐pixels; (c) applying morphological dilation (3 mm) to the segmented endocardial LA boundary and PV from MA‐WHS to extract the LA wall and PV; (d) finding the overlapped regions of the LA wall and PV and the labeled enhanced super‐pixels, and (e) labeling the other super‐pixels overlapped with LA wall and PV as nonenhancement. Details of each step are given as following:

1. Manual mouse clicks: Instead of manually drawing the boundaries of the enhanced atrial scarring regions, we asked an experienced cardiologist specialized in cardiac MRI to perform manual mouse clicks on the LGE MRI images to label the regions that they believed to be enhanced (i.e., atrial scarring tissue). This is because manual boundary drawing of enhancement on the thin LA wall is a very challenging task and subject to large inter‐ and intraobserver variances. Mouse clicks on the enhancement regions are much easier and much more efficient. The manual mouse clicks were done on the original LGE MRI images without the super‐pixel grid overlaid. This is because: (a) the mouse clicks will not be biased by super‐pixel patches and (b) the super‐pixel grid may reduce the visibility of the enhancement on LGE MRI images.
2. The coordinates of the mouse clicks were used to select the enhanced super‐pixels. Because the cardiologist performed the mouse clicks on the original LGE MRI images without having prior knowledge about the super‐pixels, we asked the cardiologist to have relatively dense mouse clicks. These mouse clicks will ensure all the enhanced regions can be included, but only one mouse click will be taken into account if multiple clicks dwell in the same super‐pixel.
3. The endocardial LA boundary and PV were extracted using our MA‐WHS method. We then applied a morphological dilation to extract the LA wall and PV assuming that the thickness of LA wall is 3 mm. The blood pool regions were extracted by a morphological erosion (5 mm) from the endocardial LA boundary, and the pixel intensities were normalized according to the mean and standard deviation of the blood pool intensities.^5^
4. We masked the selected enhanced super‐pixels [derived from step (2)] using the LA wall and PV segmentation. Only the super‐pixels having a defined overlap with the LA wall and PV segmentation were selected as enhancement for building the training data (overlapping ratio was set to ≥20%). Other super‐pixels (overlapping ratio <20%) were discarded as they were considered as enhancement from other substructures of the heart (such as the mitral valve and aorta) but not enhancement of the LA wall and PV. *It is of note that although we assumed that the LA wall thickness is 3 mm, our enhanced super‐pixels are not restricted to this wall thickness*.
5. The other super‐pixels overlapped with the LA wall and PV but not selected as enhancement were considered as nonenhancement (overlapping ratio was set to ≥20%).

By performing the five steps described above, we constructed a training dataset that contains super‐pixels labeled either enhancement or nonenhancement within the LA wall and PV.

Instead of extracting texture or shape features of these labeled super‐pixels, we computed the pixel intensity‐based features to feed to the SVM classifier. This is because the size of our super‐pixels is too small to catch enough information about texture and shape. In this study, we extracted 16 features for each super‐pixel: minimum, maximum, mean, median, standard deviation, variance, mean of the absolute deviation, median absolute deviation, coefficient of variance, skewness, kurtosis, mode, central moments, range, interquartile range, and entropy. Feature selection was done using minimum redundancy and maximum relevance method.^59^ In this study, we applied the mutual information quotient scheme.^59^ The selected features will be presented in Section 3 and will be used for the further SVM‐based classification procedure. The parameters of the SVM with a RBF kernel were optimized using cross‐validation with a grid search scheme.^60^

### 2.E. Results evaluation and validation

#### 2.E.1. Evaluation and validation of the MA‐WHS

One experienced cardiologist (>5 yr of experience and specialized in cardiac MRI) manually segmented the endocardial LA boundary and labeled the PV slice‐by‐slice in the LGE MRI images for all the patients. A second senior cardiologist (>25 yr of experience and specialized in cardiac MRI) confirmed the manual segmentation. The evaluation and validation of our MA‐WHS has been done against this manual segmentation, which is assumed to be the ground truth. We used six metrics: Dice score, Jaccard index,^61^ Precision, Negative Predictive Value (NPV), Hausdorff distance^62^, and Average Surface Distance (ASD) (defined in Table 2 for the delineated LA and PV ROIs). DICE, JACCARD, PRECISION, and Negative Predictive Value (NPV) measure the overlap (in percentage) between the manual and automatic segmentations. JACCARD is numerically more sensitive to mismatch when there is reasonably strong overlap than DICE or PRECISION. The higher the values of DICE, JACCARD, NPV, and PRECISION, the better the overall performance of the segmentation. HAUSDORFF and ASD measure the boundary distance (in mm) between two contours of segmentation. The lower the values of HAUSDORFF and ASD the better the agreement between manual delineation and fully automatic segmentation.

**Table 2:** Summary of the quantitative evaluation methods. F_Manual_: ground truth segmentation; F_Auto_: automatic segmentation; |•|: the number of pixels assigned to the segmentation; T: the total number of pixels; P_Manual_ = {p_m1_, ···, p_mn_} and P_Auto_ = {p_a1_, ···, p_an_}: two finite point sets of the two segmented contours (using the ground truth segmentation and automatic segmentation); ||•||: L_2_ norm; sup: supremum and inf: infimum. For the MA‐WHS method we used all the six evaluation metrics and for the final atrial scarring segmentation we employed DICE, JACCARD, PRECISION, and NPV

Evaluation metrics	Definition	MA‐WHS	Atrial scarring segmentation
Dice score	$\text{DICE}=\frac{2\times |F_{\mathrm{Manual}}\cap F_{\mathrm{Auto}}|}{|F_{\mathrm{Manual}}\left|+\right|F_{\mathrm{Auto}}|}$	•	•
Jaccard index	$\text{JACCARD}=\frac{|F_{\mathrm{Manual}}\cap F_{\mathrm{Auto}}|}{|F_{\mathrm{Manual}}\cup F_{\mathrm{Auto}}|}$	•	•
Precision	$\text{PRECISION}=\frac{|F_{\mathrm{Manual}}\cap F_{\mathrm{Auto}}|}{|F_{\mathrm{Auto}}|}$	•	•
Negative Predictive Value	$\text{NPV}=\frac{T-|F_{\mathrm{Manual}}\cup F_{\mathrm{Auto}}|}{T-|F_{\mathrm{Auto}}|}$	•	•
Hausdorff distance	$$\begin{array}{cc} & \text{HAUSDORFF}\left(P_{\mathrm{Manual}},P_{\mathrm{Auto}}\right)=\max\left(d\left(P_{\mathrm{Manual}},P_{\mathrm{Auto}}\right),d\left(P_{\mathrm{Auto}},P_{\mathrm{Manual}}\right)\right)\\ & \text{where}\;d\left(P_{\mathrm{Manual}},P_{\mathrm{Auto}}\right)={\text{sup}}_{p_{m}\in P_{\mathrm{Manual}}}{\text{inf}}_{p_{a}\in P_{\mathrm{Auto}}}\left|\right|p_{m}-p_{a}\left|\right|\\ & \;d\left(P_{\mathrm{Auto}},P_{\mathrm{Manual}}\right)={\text{sup}}_{p_{a}\in P_{\mathrm{Auto}}}{\text{inf}}_{p_{m}\in P_{\mathrm{Manual}}}\left|\right|p_{m}-p_{a}\left|\right| \end{array}$$	•	
Average Surface Distance	$\text{ASD}=\frac{1}{2}\left(\frac{\sum_{p_{m}\in P_{\mathrm{Manual}}}\min_{p_{a}\in P_{\mathrm{Auto}}}\|p_{m}-p_{a}\|}{\sum_{p_{m}\in P_{\mathrm{Manual}}}1}+\frac{\sum_{p_{a}\in P_{\mathrm{Auto}}}\min_{p_{m}\in P_{\mathrm{Manual}}}\left\|p_{m}-p_{a}\right\|}{\sum_{p_{a}\in P_{\mathrm{Auto}}}1}\right)$	•	

#### 2.E.2. Ground truth definition of the atrial scarring

We formed the ground truth of the enhanced atrial scarring on the LGE MRI images using the following steps:

1. Steps (1)–(4) as listed in the “SVM Based Classification” section.
2. Once the enhanced super‐pixels were extracted, they were combined to create a binary image for each slice, that is, 1 for enhanced super‐pixels and 0 for unenhanced.
3. The binary image was overlaid on the original LGE MRI images and our cardiologist performed manual corrections to create the final boundaries (ground truth) of the enhanced atrial scarring. It should be stressed here that the cardiologist used the binary image only as an initial starting point for the manual segmentation in order to reduce an otherwise very lengthy process. While this could potentially introduce a bias toward the initial binary image, the cardiologist was free to make as many edits as needed and this bias would expected to be small.

#### 2.E.3. Intra‐ and interobserver variances of the manual atrial scarring segmentation

Two cardiologists performed the manual mouse clicks based ground truth construction procedures in 8 randomly selected patients (four pre‐ and four postablation cases; 235 2D images in total) in order to determine interobserver variance. In addition, one cardiologist performed the manual mouse clicks twice at two different time points (1 month in between) to estimate the intraobserver variance. The DICE metric was used to measure the intra‐ and interobserver variances of the ground truth construction.

#### 2.E.4. Evaluation and validation of the fully automated atrial scarring segmentation

The SVM‐based classification was evaluated by: (a) leave‐one‐patient‐out cross‐validation (LOO CV), which provides an unbiased predictor and is capable of creating sufficient training data for studies with small sample size;^63^ (b) the cross‐validated classification accuracy, sensitivity, specificity, and average area under the receiver operating characteristic (ROC) curve (AUC), and (c) the balanced error rate (BER).^64^ We also applied 10‐fold CV to evaluate the robustness of our method when there are fewer manual labeled training datasets. Lastly, we divided our data into (a) a training/CV dataset (25 patients) and (b) an independent testing dataset (12 patients). In this case, we optimized SVM parameters in the training/CV dataset and then validated our method using these parameters in the independent testing dataset. This validation demonstrates the robustness of our method on an “unseen” independent testing dataset after the SVM parameters have been optimized and fixed.

For the final atrial scarring segmentation, we also performed result evaluation using DICE, JACCARD, PRECISION, and NPV measurements (Table 2). HAUSDORFF and ASD metrics were not applied because we have multiple discrete regions of enhanced atrial scarring for each LGE MRI volume.

In addition, the fibrosis extent percentage (FEP) was determined for ground truth and automatic segmentations and compared using two‐sample Wilcoxon analysis. The FEP of the atrial scarring is an important imaging biomarker for predicting the outcome of the AF treatment. FEP is defined as the volume of scar tissue as a percentage of the atrial wall volume.^15^

#### 2.E.5. Comparison study

In order to demonstrate the efficacy of our method, we also compared it with two standard methods published in previous studies:

- Simple thresholding‐based method (Thr).^26^ The threshold value for each LGE MRI volume was chosen via empirical evaluation.
- Conventional standard deviation (SD)^10^ based method (2, 4, and 6 SDs were tested).

These methods were selected as they have minimum parameter tuning and could be most accurately reproduced, and also because they are the most frequently used.

In addition, we also compared our method with state‐of‐the‐art unsupervised learning‐based clustering and graph cuts‐based methods^5^ including k‐means clustering‐based method (KM), graph cuts with k‐means clustering‐based method (KM + GC), fuzzy c‐means clustering‐based method (FC), and graph cuts with fuzzy c‐means clustering‐based method (FCM + GC). In each case, we have reproduced the method as best possible, and where tuning parameters have not been clearly defined, we have used values based on those in the original methodology publications.

We compared the atrial scarring segmentation from each method against the ground truth using the LA and PV boundaries derived from our fully automatic MA‐WHS segmentation. This was then repeated using the manually delineated LA and PV boundaries. The image intensities were normalized with respect to the mean and standard deviation of the intensities in the LA blood pool cavity,^5^ which were extracted by a morphological erosion (5 mm) from the endocardial LA boundary.

For comparison studies, statistical significances were given by two‐sample Wilcoxon rank‐sum test.

## 3 Results

### 3.A. Whole heart segmentation results

**Figure 2:**
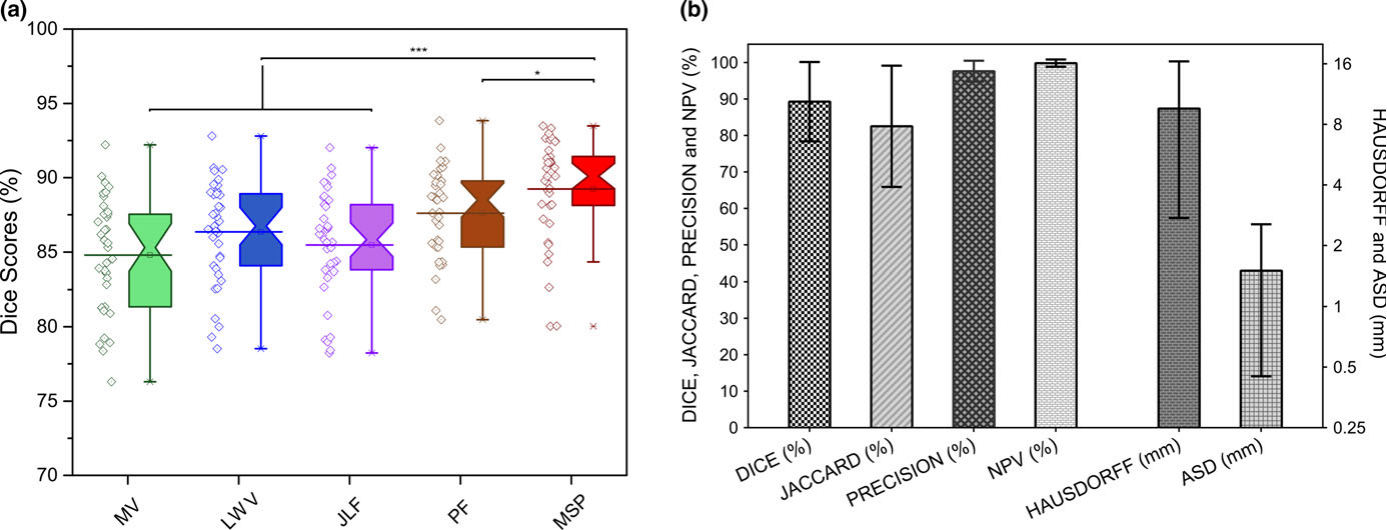
(a) Comparison results (Dice scores of the WHS) of using different label fusion algorithms. (“*” = *P* < 0.05 and “***” = *P* < 0.0005; statistical significances were given by two‐sample Wilcoxon rank‐sum test). Abbreviations: MV, majority vote; LWV, local weighted voting; JLF, joint label fusion; PF, patch fusion one scale; MSP, multiscale patch. (b) quantitative comparison of MA‐WHS compared to manually determined ground truth. [Color figure can be viewed at http://wileyonlinelibrary.com]

Figure 2(a) shows the comparison results of the Dice scores using the different label fusion schemes, for example, by majority vote (MV), by local weighted voting (LWV),^37^ by joint label fusion (JLF),^43^ by patch fusion one scale (PF), and by our proposed MSP. The MSP was significantly better than the other label fusion schemes (*P* < 0.05). Figure 2(b) shows the quantitative results of this MA‐WHS method compared to the ground truth.

### 3.B. Fully automated atrial scarring segmentation results

#### 3.B.1. Intra‐ and interobserver variances of the ground truth construction

**Figure 3:**
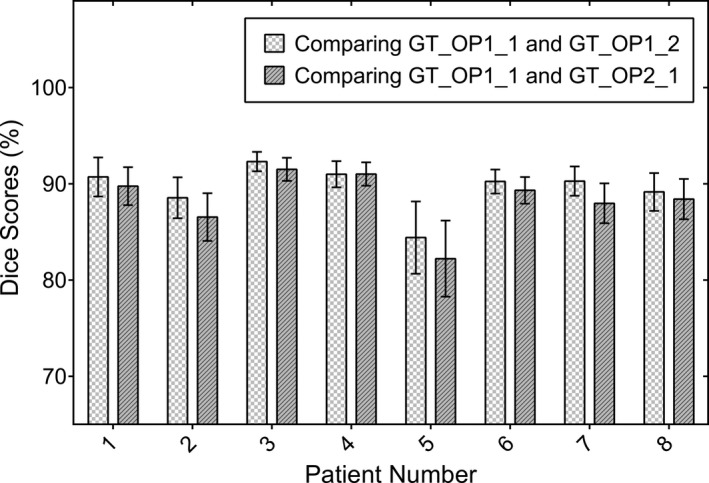
Comparison of the manual delineations (ground truth) of the enhanced atrial scarring to demonstrate the inter‐ and intraobserver variances for eight randomly selected patient cases (235 2D slices in total). The mean and standard deviation (error bars) are shown. Abbreviations: GT_OP1_1: ground truth/operator 1/time point 1; GT_OP1_2: ground truth/operator 1/time point 2; GT_OP2_1: ground truth/operator 2/time point 1.

Figure 3 demonstrates the intra‐ and interobserver variances of the manual atrial scarring delineation. Both intra‐ and interobserver agreement are very good (mean DICE scores ranging from 86% to 92% and from 83% to 91%, respectively) and confirm the suitability of the ground truth reconstructions for evaluation of the atrial scarring segmentation algorithms.

#### 3.B.2. Evaluation and validation results of the fully automated atrial scarring segmentation

After minimum redundancy and maximum relevance based feature selection, 3 of 16 features (minimum, mean and standard deviation) were selected and used in building the SVM model. Table 3 tabulates the SVM classification results of distinguishing enhanced atrial scarring regions from nonenhanced tissues. Using LOO CV, we obtained 88% accuracy and 0.16 BER. ROC analysis shows an AUC of 0.91. In terms of the final segmentation accuracy, we achieved mean DICE of 79%. Compared to manual ground truth construction that took 25–50 min per patient case, the SVM‐based prediction only took 5.1 ± 0.7 s to segment one patient case, while for a single loop of the LOO CV the training on 36 patients took 64.2 ± 4.6 min (~1.8 min per patient case). All experiments were performed using a Windows 7 workstation with six‐core 1.9 GHz Intel^®^ Xeon^®^ E5‐2609v3/64 GB RAM.

**Table 3:** Quantitative evaluation of the atrial scarring segmentation using LOO CV, 10‐fold CV, and training/CV with separate testing. The SVM based classification was evaluated using accuracy, sensitivity, specificity, BER, and AUC. The final segmentation was evaluated against the ground truth using Precision, NPV, Jaccard index and Dice score. Abbreviations: LOO, leave‐one‐patient‐out; CV, cross‐validation; BER, balanced error rate; AUC, area under curve; NPV, negative predictive value

Validation method		Accuracy (%)	Sensitivity (%)	Specificity (%)	BER	AUC	Precision (%)	NPV (%)	Jaccard Index (%)	Dice Score (%)
LOO CV (37 patients)		88	90	79	0.16	0.91	81 ± 9	99 ± 1	65 ± 6	79 ± 5
10‐fold CV (37 patients)		88	96	62	0.21	0.91	86 ± 4	99 ± 2	56 ± 3	72 ± 2
Training/CV + separate testing	LOO CV (25 patients)	87	89	79	0.16	0.91	80 ± 10	99 ± 1	66 ± 6	79 ± 5
	Separate testing (12 patients)	86	92	64	0.22	0.88	77 ± 7	99 ± 1	56 ± 7	71 ± 7

Ten‐fold cross‐validation obtained similar accuracy. For the validation on the separated independent testing datasets, the fixed SVM model was blindly built. Compared to LOO CV on 37 datasets we achieved similar accuracy (86%) and sensitivity (92%), but lower specificity (64%) and mean DICE (71%). This validation using separate testing datasets showed that our method can still perform well while fixing the classification model and using it to segment the new input data.

**Figure 4:**
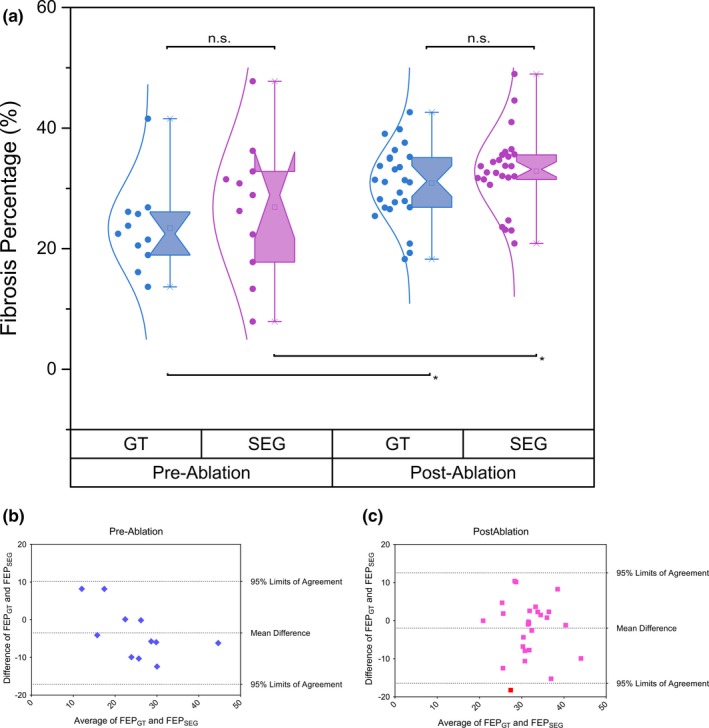
(a) The percentage of fibrosis extent calculated using the ground truth segmentation (GT in blue boxplots) and using our fully automatic segmentation pipeline (SEG in purple boxplots) for both the preablation cases and the postablation cases, respectively. (“*” = *P* < 0.05 and “n.s.” means no significant difference between two groups; statistical significances were given by two‐sample Wilcoxon rank‐sum test). Abbreviations: GT, ground truth segmentation; SEG, segmentation using our fully automatic pipeline. (b)–(c) Bland–Altman analysis of the measurements of fibrosis extent derived by using the ground truth segmentation (FEP_GT_) and our fully automatic segmentation pipeline (FEP_SEG_). Abbreviations: GT, ground truth segmentation; SEG, segmentation using our fully automatic pipeline; FEP, fibrosis extent percentage. [Color figure can be viewed at http://wileyonlinelibrary.com]

Using our fully automatic pipeline, the measured native (preablation) fibrosis associated with AF was 26.9 ± 11.2% compared to 32.8 ± 6.4% for the postablation cases [Fig. 4(a)]. There was no significant difference found for the fibrosis extent derived using the ground truth segmentation of the atrial scarring (23.4 ± 7.3% for the preablation cases and 30.9 ± 6.1% for the postablation cases). The high fibrosis extent measured by both techniques in the preablation patients reflects the extensive native fibrosis due to atrial remodeling in this patient cohort with long‐standing persistent AF. Both our fully automatic pipeline and the ground truth segmentation found significant differences in fibrosis extent between pre‐ and postablation cases [Fig. 4(a)]. Bland–Altman analyses are shown in Figs. 4(b) and 4(c).

**Figure 5:**
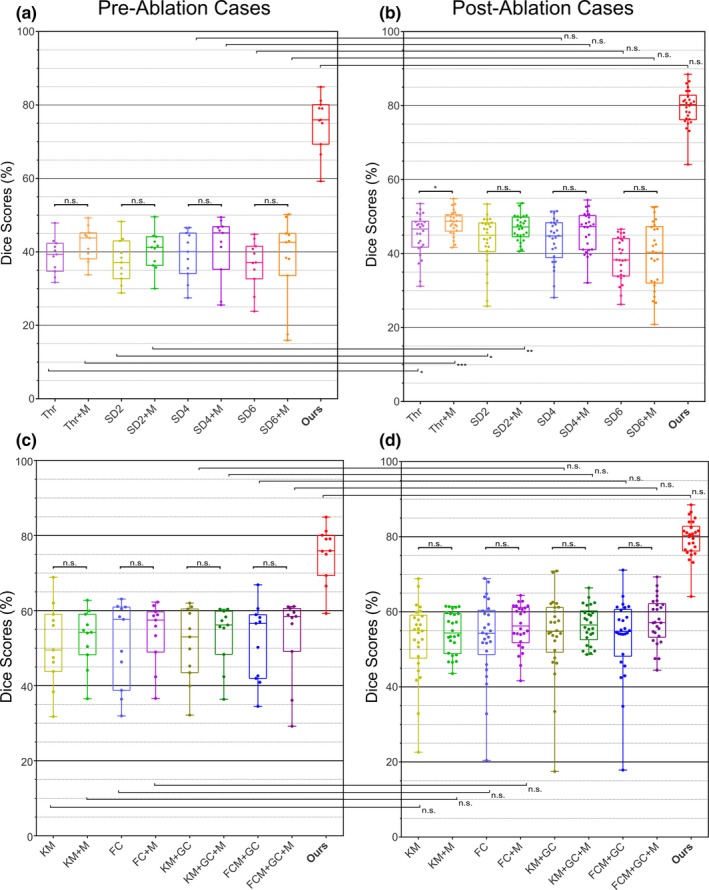
Comparison results with conventional atrial scarring segmentation methods using DICE for (a) and (c) preablation and (b) and (d) postablation cases. (“*” = *P* < 0.05, “**” = *P* < 0.005, “***” = *P* < 0.0005, and “n.s.” means no significant difference between two groups; statistical significances were given by two‐sample Wilcoxon rank‐sum test). Abbreviations: Thr, simple thresholding‐based method with MA‐WHS‐derived LA + PV; SD(x), conventional standard deviation method (x = 2, 4 and 6 SDs were tested) with MA‐WHS‐derived LA + PV; KM, k‐means clustering‐based method with MA‐WHS‐derived LA + PV; FC, fuzzy C‐means clustering‐based method with MA‐WHS‐derived LA + PV; KM + GC, graph cuts and k‐means clustering with MA‐WHS‐derived LA + PV; FCM + GC, graph cuts and fuzzy C‐means clustering with MA‐WHS‐derived LA + PV; (x) + M, different methods with manual delineated LA + PV; MA‐WHS, multiatlas whole heart segmentation; LA + PV, left atrium and pulmonary veins. [Color figure can be viewed at http://wileyonlinelibrary.com]

Figure 5 shows the results of the comparison study in preablation [Figs. 5(a) and 5(c)] and postablation [Figs. 5(b) and 5(d)] cases with our method (red bars) working equally well in both (median DICE score 80% for the postablation cases vs. median DICE score 76% for the pre‐ablation cases, *P* = 0.087). Overall, the atrial scarring segmentation results obtained using our method outperformed the simple thresholding and conventional standard deviation methods significantly [Figs. 5(a) and 5(b)]. While, as expected, DICE scores for the state‐of‐the‐art unsupervised learning based clustering and graph‐cuts based methods were better than thresholding and standard deviation based methods [Figs. 5(c) and 5(d)], our method again showed superior results.

**Figure 6:**
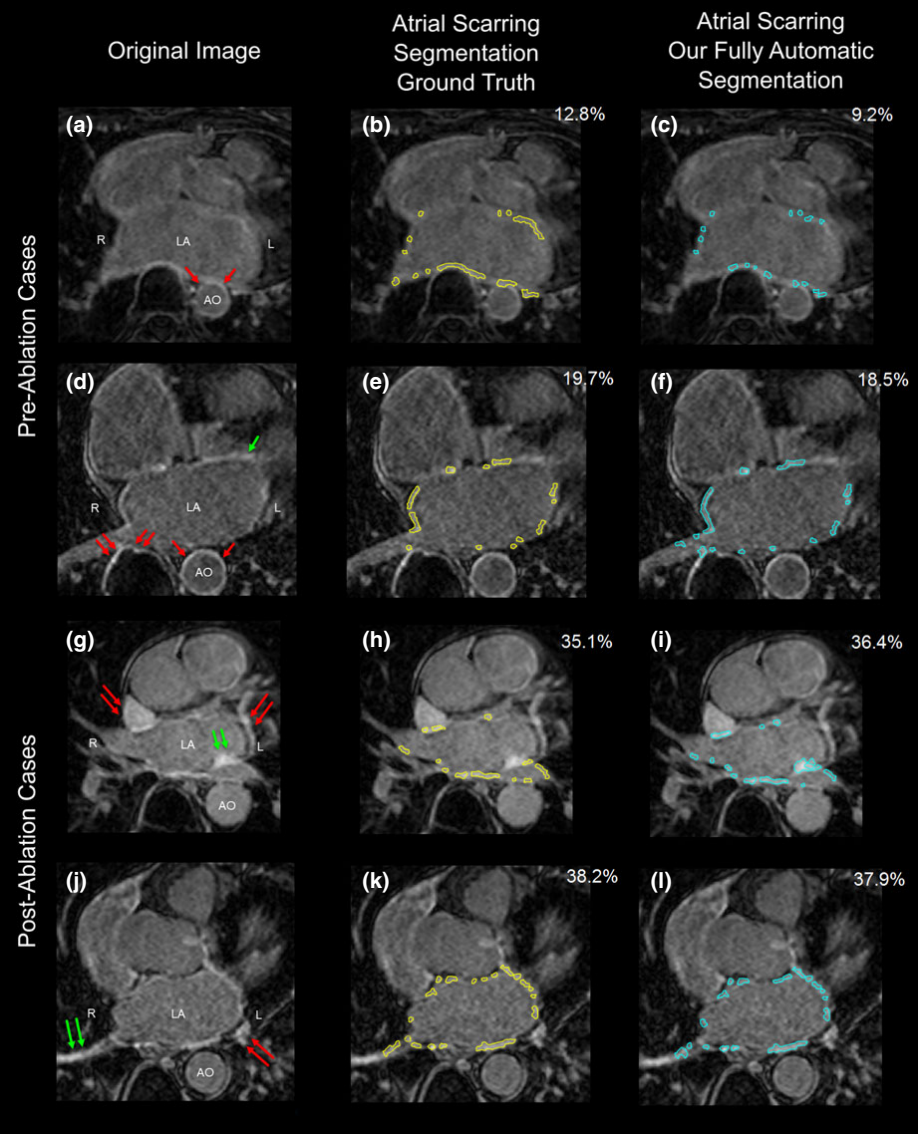
Final atrial scarring segmentation results of two preablation cases (a–c) and (d–f) and two postablation cases (g–i) and (j–l). (a), (d), (g), and (j) Original LGE MRI images; (b), (e), (h), and (k) Ground truth of the atrial scarring segmentation; (c), (f), (i), and (l) Results of our fully automatic atrial scarring segmentation. Single red arrows in (a) and (d) show the enhancement of the AO wall. Double red arrows in (d), (g), and (j) show enhanced regions outside the heart, in other substructures inside the heart or in surrounding fat tissue. Single green arrow in (d) shows the enhanced artifacts of the mitral valve. Double green arrows in (g) show some enhancement that, in retrospect, our cardiology experts agreed should have been included in the ground truth labeling procedure, but was found using our fully automatic segmentation. And double green arrows in (j) show the enhancement due to the navigator beam and blood flow. Abbreviations: LA, left atrium; AO, aorta; L, left; R, right. [Color figure can be viewed at http://wileyonlinelibrary.com]

Figure 6 shows our final atrial scarring segmentation results compared to the ground truth in two preablation and two postablation patients. In each case, the figure shows a single slice of a 3D LGE dataset (typically 64 slices) while the FEP values quoted are for the full 3D study. The segmentation results have been derived from the LOO CV (i.e., training on 36 datasets and making prediction on the one dataset that has been left). For the first preablation case [Figs. 6(a)–6(c)], the automatic segmentation slightly underestimates the enhancement (FEP 9.2% compared to 12.8%). Segmentation of the second preablation case [Figs. 6(d)–6(f)] shows clear accordance compared to the ground truth despite some slight overestimation near the right inferior pulmonary vein. Segmentation of both postablation cases exhibit good agreement with the ground truth [Figs. 6(i) vs. 6(h) and 6(l) vs. 6(k)].

## 4 Discussion

We have developed a novel fully automatic segmentation pipeline to detect atrial scarring in LGE MRI images and validated it against manual ground truth segmentation by experienced cardiologists.

Accurate knowledge of native and previous ablation scarring using LGE MRI may be used to stratify patients and guide ablation treatment, ultimately reducing the need for repeat procedures.^5^ Gadolinium‐based contrast agent is contraindicated in patients with severe renal impairment due to an increased risk of developing nephrogenic systemic fibrosis, a rare but serious disease. In addition, there have been recent reports describing the retention of gadolinium in the body for up to several months after administration, even in patients with normal kidney function. This has led to the Federal Drugs Administration (FDA) issuing a safety communication and while no direct association has been found between these deposits and adverse health effects in patients with normal kidney function, it has announced that a new class warning and safety measures will be required to appear in the labeling for gadolinium contrast agents used in MRI. Consideration should be given to the use of macrocyclic gadolinium agents, such as gadobutrol (used in this study), rather than linear agents as they have reduced and shorter retention properties. The FDA concludes that the benefit of all approved gadolinium contrast agents continues to outweigh potential risks and that healthcare professionals should not avoid or defer necessary examinations.^65^

Segmentation of the atrial scarring from LGE MRI images is very challenging. This is not only because the atrial scarring is difficult to distinguish in the thin LA wall but also because the image quality can be poor due to motion artifacts, noise contamination and contrast agent wash‐out during the long acquisition. Moreover, the enhancement from the surrounding tissues and enhanced blood flow can result in increased false positives. However, most of these confounding enhancement regions can be distinguished subject to accurate heart anatomy delineation using our MA‐WHS [Figs. 6(c), 6(f), 6(i), and 6(l)].

The minimum redundancy and maximum relevance method has selected three simple but effective features for our further SVM classification on SLIC segmented super‐pixels, that is, the mean, the standard deviation, and the min of the super‐pixels. The feature “mean” corresponded to a simple thresholding on the super‐pixel intensity values and the feature “standard deviation” quantified local intensity variations in the scar and healthy regions. The feature “min” was selected as a strong discriminator due to the fact that in the enhanced atrial scarring regions the “min” intensity values are much higher than the “min” intensity values of the normal regions. However, the feature “max” was not selected as a discriminator as for some labeled normal regions, there may be relatively high pixel intensities that could be false positives.

There are limitations of the current work. The fast and irregular heart rate in patients prior to ablation resulted in only 11 (65%) preablation studies having good enough quality to be included in this study. Together with 26 (92%) postablation studies, our total number of datasets was limited to 37. The low number of preablation studies included is an indication of how difficult it is to scan patients in AF. However, developments in MRI acquisition strategies (beat‐to‐beat changes in inversion time and intelligent arrhythmia rejection schemes) are likely to improve this. To tackle the problem of having limited patient data, LOO CV was performed to achieve an unbiased predictor for limited datasets.^66^, ^67^

As in other studies,^5^ we have validated our technique against a manually segmented ground truth. In our case, to reduce a very lengthy process, the starting point for this was an automatically derived binary image which the cardiologist was free to edit as much as possible. While this could introduce some bias into the ground truth, this is expected to be small and the methodology had both low inter‐ and intraoperator variance. However, even with experienced cardiologists and consensus agreement, this is subjective, and regions of enhancement can be missed [Fig. 6(g)], but they can still be found using our fully automatic segmentation pipeline. EAM may provide a more objective ground truth but is not straightforward due to issues relating to sampling point density and distribution, contact force which affects the measured voltage, voltage thresholds for delineating scar tissue, and registration and deformation of the mapped space in the EP lab to the LGE MRI study.

In this study, we compared with the simple thresholding and conventional standard deviation based methods and also a number of advanced unsupervised learning based clustering and graph cuts‐based methods.^5^ Although superior performance has been achieved using our propose method, it is of note that our patient cohort is different from that in which these comparison algorithms were optimized and tested. When compared to manual segmentation (ground truth) in postablation scans, these standard techniques gave median DICE of 38%–48% while our fully automatic technique achieved a median DICE of 80%. The results that we obtained here with the standard techniques are similar to those reported with these same techniques in the benchmarking study described in Ref. [5] while the latter score is similar to the best‐performing methods reported in that same study. Moreover, performance of the state‐of‐the‐art unsupervised learning based methods were better than thresholding and standard deviation‐based methods [Figs. 5(c) and 5(d)], but our method again showed superior results that might be attributed to the fact that our proposed method is supervised, and therefore have obtained more useful information from the manually labeled data. Of note is that in the benchmarking study, the variances of all of the techniques tested are large while in our manuscript, the results are more consistent with a relatively small variance (boxplot in red as seen in Fig. 5). This may be due to our patient cohort being more tightly defined while in the previous study, datasets were analyzed from patients at multiple institutions using a variety of imaging protocols.

## 5 Conclusions

To the best of our knowledge, this is the first study that developed a fully automatic segmentation pipeline for atrial scarring segmentation with quantitative validation for LGE MRI scans. The proposed pipeline has demonstrated an effective and efficient way to objectively segment and assess the atrial scarring. Our validation results have shown that both our MA‐WHS and super‐pixel classification‐based atrial scarring segmentation have obtained satisfactory accuracy. The current study was performed using real clinical data, and we can envisage an integration of our pipeline to clinical routines. In so doing, a patient‐specific LA and PV geometry model and an objective atrial scarring segmentation can be obtained rapidly for individual AF patients without manual processing.

## Conflict of interest

The authors have no conflicts to disclose.

## Supporting information


**Appendix S1.** Supporting material.
