# Supplementary material for: Fully automatic segmentation and objective assessment of atrial scars for long‐standing persistent atrial fibrillation patients using late gadolinium‐enhanced MRI
Source: Med Phys. 2018 Mar 15;45(4):1562–76. doi: 10.1002/mp.12832 (PMC5969251; doi:10.1002/mp.12832)
Supplement: Supplementary file 1 — Appendix S1. Supporting material. [file MP-45-1562-s001.docx]

# Supporting Material

# for

Fully Automatic Segmentation and Objective Assessment of Atrial Scars for Longstanding Persistent Atrial Fibrillation Patients Using Late Gadolinium-Enhanced MRI

*A1. Support Vector Machines (SVM)*

After SLIC segmentation, we proposed to use Support Vector Machines (SVM) to classify the over-segmented super-pixels into enhanced atrial scarring regions and non-enhanced tissues. SVM provide a powerful technique for supervised binary classiﬁcation [1]. SVM predictions depend on a subset of training data (i.e., the support vectors), and find the hyperplane with largest margin between the two classes [2]. This is obtained by solving the following optimisation problem,

1. $\min_{w,b,\zeta} \frac{1}{2}w^{T}w+\rho\sum_{i=1}^{l} \zeta_{i}$,

subject to $y_{i}\left( w^{T}\emptyset\left( x_{i} \right)+b \right)\geq1-\zeta_{i}$ and $\zeta_{i}\geq0$,

in which ${(x}_{i},y_{i}), i=1,2,\ldots,l$ is the instance-label pairs of the given training dataset [3]. Here $\left\langle w,x \right\rangle+b=0$ defines the separating hyperplane for $b$∊$\mathbb{R}$ is real. Furthermore, $L_{1}$-norm based formulation of the soft margins was applied by adding slack variables $\zeta_{i}$ and a penalty parameter $\rho$, which is known as the box constraint for the soft margin. In addition, ${K(x_{i},x_{j})\equiv\emptyset\left( x_{i} \right)}^{T}\emptyset\left( x_{j} \right)$, is called the kernel function. In this study, we used a nonlinear Gaussian Radial Basis Function (RBF) kernel $K\left( x_{i},x_{j} \right)=exp(-\gamma\left\| x_{i}-x_{j} \right\|^{2})$ with scaling-factor, $\gamma>0$, to map feature vectors into a nonlinear feature space where an optimal hyperplane was constructed to separate two different classes, i.e., enhancement and non-enhancement. The parameters of the SVM with a RBF kernel (i.e., $\rho$ and $\gamma)$ were optimised using cross-validation with a grid search scheme [3]. In this study we firstly used coarse $11\times11$ ‘grid’ using $\rho=2^{-10}, 2^{-8}, \ldots, 2^{8}, 2^{10}$ and $\gamma=2^{-10}, 2^{-8}, \ldots, 2^{8}, 2^{10}$, and then with a fine $3\times3$ ‘grid’ $\rho=2^{8}, 2^{8.5}, 2^{9}$ and $\gamma=2^{2}, 2^{2.5}, 2^{3}$. The optimisation showed that the best classification was achieved when $\rho=2^{8.5}$ and $\gamma=2^{2.5}$.

**REFERENCES FOR THE SUPPORTING MATERIAL**

1. Cristianini, N., Shawe-Taylor, J.: An Introduction to Support Vector Machines and Other Kernel-based Learning Methods. Cambridge University Press (2000).

2. Murphy, K.: Machine Learning: A Probabilistic Perspective. MIT Press (2012).

3. Hsu, C., Chang, C., Lin, C.: A practical guide to support vector classification. (2003).
